# Whole-Exome Sequencing Uncovers Novel Causative Variants and Additional Findings in Three Patients Affected by Glycogen Storage Disease Type VI and Fanconi−Bickel Syndrome

**DOI:** 10.3389/fgene.2020.601566

**Published:** 2021-01-11

**Authors:** Maryam Eghbali, Kiyana Sadat Fatemi, Shadab Salehpour, Maryam Abiri, Hassan Saei, Saeed Talebi, Nasrin Alipour Olyaei, Vahid Reza Yassaee, Mohammad Hossein Modarressi

**Affiliations:** ^1^Department of Medical Genetics, Faculty of Medicine, Tehran University of Medical Sciences, Tehran, Iran; ^2^Dr. Zenali’s Medical Genetics Laboratory, Kawsar Human Genetics Research Center, Tehran, Iran; ^3^Genomic Research Center, Shahid Beheshti University of Medical Sciences, Tehran, Iran; ^4^Department of Pediatric Endocrinology and Metabolism, Mofid Children’s Hospital, Shahid Beheshti University of Medical Sciences, Tehran, Iran; ^5^Shahid Akbarabadi Clinical Research Development Unit, Iran University of Medical Sciences, Tehran, Iran; ^6^Department of Medical Genetics and Molecular Biology, Faculty of Medicine, Iran University of Medical Sciences, Tehran, Iran

**Keywords:** glycogen storage diseases, whole-exome sequencing, novel causative variants, secondary/incidental findings, pharmacogenetic variants

## Abstract

Glycogen storage diseases (GSDs) are the heterogeneous group of disorders caused by mutations in at least 30 different genes. Different types of GSDs, especially liver GSDs, take overlapping symptoms and can be clinically indistinguishable. This survey evaluated the use of whole-exome sequencing (WES) for the genetic analysis of the liver GSD-suspected patients in three unrelated families. An in-house filtering pipeline was used to assess rare pathogenic variants in GSD-associated genes, autosomal recessive/mendelian disorder genes (carrier status for genetic counseling subjects), and the ACMG’s list of 59 actionable genes. For the interpretation of the causative variants and the incidental/secondary findings, ACMG guidelines were applied. Additionally, we have explored PharmGKB class IA/IB pharmacogenetic variants. The segregation analysis was performed using Sanger sequencing for the novel causative variants. Bioinformatics analysis of the exome data in three individuals revealed three novel homozygous causative variants in the GSD-associated genes. The first variant, c.298_307delATGATCAACC in PYGL gene has related to HERS disease (GSD VI). Both variants of c.1043dupT and c.613-1G > C in SLC2A2 gene have been associated with Fanconi-Bickel syndrome (GSDXI). Eight pathogenic/likely pathogenic medical actionable findings in Mendelian disease genes and 10 pharmacogenetic variants with underlying drug response phenotypes have been identified. No known/expected pathogenic variants were detected in the ACMG’s list of 59 actionable genes. The logical filtering steps can help in finding other medical actionable secondary/incidental findings as well as effectively identifying the causative variants in heterogeneous conditions such as GSDs. Three novel variants related to GSD genes recognized in liver GSD-suspected patients with early infantile and childhood-age onset.

## Introduction

Glycogen storage diseases (GSDs) are a group of rare inborn errors of metabolism disorders caused by errors of specific enzymes involving in glycogen degradation and synthesis. Based on the enzyme deficiency and the involved tissue/s, GSDs are classified into two major groups: liver GSDs and muscle GSDs. Other organs such as the heart, and sometimes the central nervous system can be commonly affected. There are nearly thirty types of GSD, in which, the most common types, i.e., II, IIIa, V, VII, IXd, X, XII, XIII, and XIV involve muscles (skeletal muscle or heart) and types I, III, 0, XI, IX, VI, and IV mainly affect the liver (Wang et al., 2012).

In general, the frequency of GSD is estimated to be in 2,000–43,000 live births. The clinical presentation is wide-range from exercise intolerance, muscle- and heart-disturbance, and sometimes rhabdomyolysis. Hepatomegaly and hypoglycemia are the shared features of liver GSD types. GSDs have variations in the age of onset, the severity of symptoms, morbidity, mortality, and prognosis, which is associated with the specific causal variant. Clinical symptoms may vary from severe (neonatal and infantile) to almost asymptomatic or only exercise intolerance or underestimate due to mild symptoms (Kishnani et al., 2010, 2014; Laforet et al., 2012; Wang et al., 2012).

Different types of GSDs especially liver GSDs are clinically indistinguishable with overlapping symptoms, even with the occurrence of the same enzyme/mutation. The clinical symptoms of GSD associated with the liver, such as GSD I, GSD III, GSD IXa, and GSD XI, are similar to each other, while the management/treatment may be different. Direct mutation detection is the best method to confirm the clinical diagnosis and classifying different forms of GSDs. Whole-exome sequencing (WES) is an invaluable and essential tool to determine the causative variants with accuracy and efficacy in heterogeneous disorders such as GSDs.

Glycogen storage disease type VI (GSD-VI) and Fanconi-Bickel syndrome (FBS) are the two types of GSDs that are investigated in this study. GSD VI is a clinically and genetically heterogeneous group of disorders with hepatomegaly, early fasting hypoglycemia, growth retardation, and hyperlipidemia (Guillam et al., 1997; Santer et al., 2002). This rare autosomal recessive disorder with a frequency of 1:100,000 presents a relatively mild disorder in infancy and childhood (Burwinkel et al., 1998; Chang et al., 1998; Aeppli et al., 2020). It is caused by compound heterozygous or homozygous mutations of the PYGL gene (deficiency of liver phosphorylase) (Luo et al., 2020). PYGL gene mutations inhibit the normal function of the liver glycogen phosphorylase enzyme. This enzyme cleaves alpha 1,4-glycosidic bonds to release glucose 1-phosphate from glycogen (Burwinkel et al., 1998; Chang et al., 1998). The PYGL gene has 20 exons and is located on chromosome 14. About 40 different mutations in the PYGL gene have been reported (in the Human Gene Mutation Database^1^), containing point mutations, splice-site mutations, and deletions (Roscher et al., 2014).

Fanconi-Bickel syndrome also known as GSD type XI (GSD XI), a rare disorder of carbohydrate metabolism with an autosomal recessive mode of inheritance, was described first by Fanconi and Bickel (1949). It is caused by missense, nonsense, frame-shift (fs), in-frame indels, splice site, and compound heterozygous mutations in the GLUT2 (SLC2A2) gene. More than 100 cases of FBS with 70 specific mutations in this gene have been identified so far (Sharari et al., 2020). SLC2A2 gene mapped on chromosome 3q26.1-26.3 and encoded facilitative glucose transporter protein 2 (GLUT2). SLC2A2 gene contains 11 exons and codes 524 amino acids (aa) which the transmembrane segments of 9–12 show a major part in the typical affinity for glucose. GLUT2 protein is expressed in hepatocytes, enterocytes, pancreatic β-cells, and renal tubular cells (Sakamoto et al., 2000; Sharari et al., 2020). Mutations in the SLC2A2 gene are characterized by the accumulation of glycogen, specifically in the liver and kidneys, failure to thrive, fasting hypoglycemia, short stature, tubular nephropathy (glucosuria, proteinuria, phosphaturia, bicarbonate wasting, and aminoaciduria), rickets, and growth retardation. Additionally, FBS also has been reported with phenotypic heterogeneity and variation in clinical severity from mild presentation to overt diabetes mellitus (Şeker-Yılmaz et al., 2017; Sharari et al., 2020).

In the present study, we performed WES to uncover the disease-causing variants in three Iranian suspected liver GSD patients. We assessed exomes by an in-house filtering pipeline for rare likely pathogenic and pathogenic variants in 30 GSD-associated genes for identifying causative variants associated with the patient’s phenotype. To discover incidental/secondary findings, we searched rare coding variants in other genes associated with Mendelian diseases (carrier status for genetic counseling subjects) in the Online Mendelian Inheritance in Man (OMIM) database or on in the ACMG’s list of 59 actionable genes (associated with the preventable or curable disease in childhood/adulthood) (Green et al., 2013; Kalia et al., 2017). In addition, secondary pharmacogenetic variants in the Pharmacogenomics Knowledgebase (PharmGKB class IA/IB) and ClinVar were explored.

## Materials and Methods

### Subjects and Ethics Statement

Three patients (from three unrelated consanguineous families) with the clinical diagnosis of hepatic/liver GSD were recruited from the Taleqani Hospital and the Genomic Research Center in Tehran, Iran, for the present study. Inclusion criteria were based on clinical presentations of hepatomegaly, and biochemical laboratory tests such as hypoglycemia, hypertriglyceridemia, hyperlactatemia, hyperuricemia, and elevated aspartate aminotransferase (AST) or alanine transaminase (ALT). Assessment of serum phosphorus, serum calcium, and urine analysis was also performed. WES was performed on three affected GSD patients and identified variants were confirmed by Sanger sequencing in their parents. Genetic counseling was done with an expert genetic counselor and written informed consent was achieved from all subjects and/or their parents. The study was approved by the Ethical Committee of the Tehran University of Medical Sciences.

### Whole-Exome Sequencing

Peripheral blood samples of the three patients and their parents were used to extract genomic DNA using the Salting out procedure (Mwer et al., 1988). The patients (IV-2, IV-1, and V-3) were subjected to WES using Agilent SureSelect V5 Target Enrichment Kit. The enriched library was sequenced on an Illumina HighSeq4000 platform. Paired-end 101-bp sequence reads were mapped to the UCSC human reference genome (GRCh37/hg19 assembly) by Burrows-Wheeler Aligner (BWA) (Li and Durbin, 2010). Duplicate and low-quality reads (QBase < 20) were removed. Samtools (Li et al., 2009) was used for sorting and indexing bam files. To call single nucleotide variants (SNVs) and short insertions or deletions (Indels), Genome Analysis Tool Kit (GATK) software package^2^ were used. The VCF data were submitted to the Wannovar tool^3^ for variant annotation.

### Data Analysis (Variant Filtering)

**FIGURE 1:**
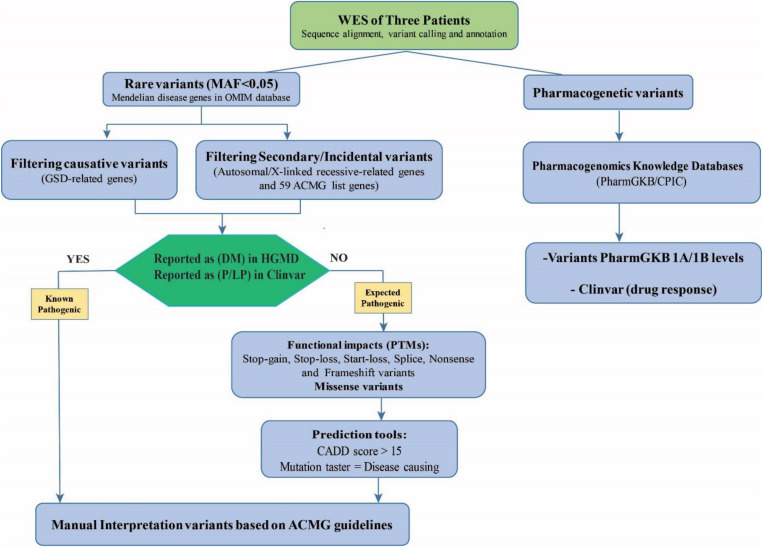
The workflow filtering of the causative variants and the secondary actionable findings in the patients for the WES results. The assessment of WES data consists of filtering for rare known pathogenic (P) and likely pathogenic (LP) variants in genes in the OMIM database including the GSD-associated genes, the ACMG’s list of 59 genes, and other genes associated with mendelian diseases (autosomal/X-linked recessive-related genes). For prioritizing expected pathogenic variants, protein-truncating variants (PTMs) and missense variants were selected and bioinformatics prediction tools were used to evaluate the potential impact and pathogenicity of the candidate variants (CADD score > 15 and mutation taster = disease causing). The identified variants were categorized and interpreted according to the ACMG-AMP 2015 Standards and Guidelines. Besides, secondary pharmacogenetic variants in the Pharmacogenomics Knowledgebase (PharmGKB class IA/IB) and ClinVar database were explored.

An in-house filtering pipeline was conducted to detect the causative variants. The stepwise approach for data analysis is shown in Figure 1. The VCF annotated files were filtered to catch rare pathogenic (P) and likely pathogenic (LP) variants in OMIM genes; including the GSD-associated genes, the list of 59 ACMG genes (Green et al., 2013; Kalia et al., 2017), and other genes associated with Mendelian diseases (the heterozygote variants/carrier status for genetic counseling). Secondary pharmacogenetic variants were also investigated.

Common variants were excluded with minor allele frequency > 0.05 in comparison within dbSNP^4^, 1000 Genomes database^5^, NHLBI Exome Sequencing^6^, ExAC database^7^, and gnomAD database^8^. Then intronic, synonymous, upstream/downstream variants were removed but the 20–30 bp flanking each exon was included in annotated files.

The remaining SNVs and Indels variants were prioritized as follows: (a) known/unknown loss-of-function mutations (non-sense, splice site, frameshift, in-frameshift variants, start-loss) and missense mutations were selected and checked in ClinVar^9^ and HGMD^10^; (b) Bioinformatics prediction assessments to evaluate the potential impact and pathogenicity of the candidate causative variants on the function or structure of the protein and conservation were directed by SIFT (Sim et al., 2012; Vaser et al., 2016), PolyPhen2 (Adzhubei et al., 2013), Mutation Taster (Schwarz et al., 2014), Combined Annotation Dependent Depletion (CADD) (Kircher et al., 2014), DANN score (Quang et al., 2015), and GERP score (Davydov et al., 2010); (c) the Varsome and Interval tools which are based on ACMG-AMP 2015 Standards and Guidelines (Richards et al., 2015) were used to interpret variants (Li and Wang, 2017; Kopanos et al., 2019).

To evaluate pharmacogenetic variants and drug efficacy, we used the Clinical Pharmacogenetics Implementation Consortium (CPIC), the PharmGKB database (1A/1B categories), and drug responses based on ClinVar database. PGKB 1A/1B levels are the substantive evidence for clinical relevance. Level 1A represents annotation for a variant-drug combination in a CPIC and Level 1B shows annotation for a variant-drug combination with a replicated association in more than one cohort with significant *p*-values.

### Sanger Sequencing for Validation of the Identified Mutations

Following data filtering, the identified causative variants were confirmed by conventional Sanger sequencing in DNA samples of the patients and their parents. For this purpose, specific primers were designed with the Primer3 website^11^ and the amplicons were directed to sequencing with ABI 3500 Genetic Analyzer (Applied Biosystems, Foster City, CA, United States). The reference sequences were used from the Ensemble^12^, and the data analysis of sequencing results was done with Chromas v2.33 software.

## Results

### Clinical Features of Patients

**FIGURE 2:**
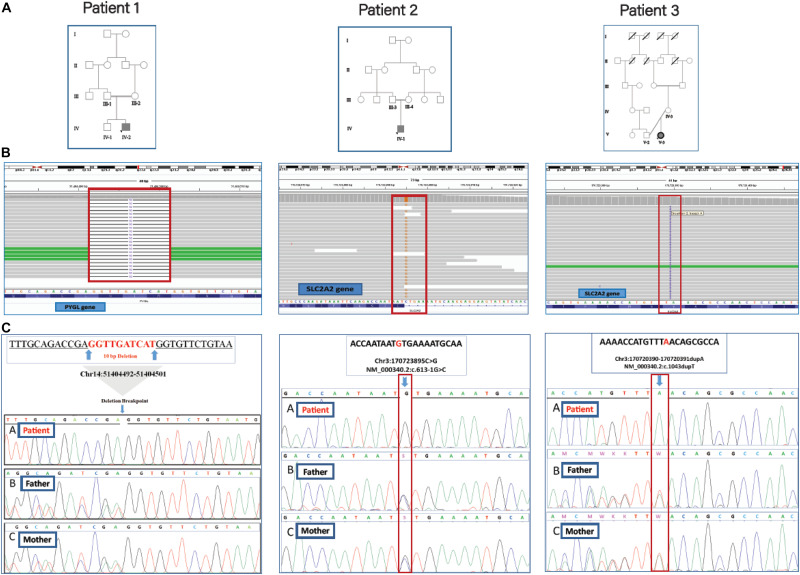
The family trees, BAM interfaces, and segregation analysis results. **(A)** The pedigrees of patients 1, 2, and 3 were shown in which their parents are consanguine. **(B)** The WES reads were visualized by using the Integrative Genomics Viewer (IGV). **(C)** Sanger sequencing confirmation for the causative variants in homozygous patients and unaffected heterozygous parents.

The proband (IV-2), a 5-year old boy (Figure 2) was a consequence of a consanguineous marriage. He tended to grow slower than his elder brother and midparental height. However, at 5 years old, compared to the growth charts, his weight (17.4 kg) and height (107 cm) were within the normal ranges for his sex and age. There was also a history of mild delays in the development of speech and motor skills, such as sitting, standing, or walking compared to his brother. Other than a history of neonatal jaundice, he has no problems until he was 3 years old when he experienced a fit after overnight fasting on an early holiday morning. At that time he was admitted to the hospital with a primary diagnosis of “ketotic hypoglycemia of childhood” as there were low blood sugar, mild metabolic acidosis, slightly elevated blood lactate, and positive urine ketosis. On the other hand, physical and ultrasound exams revealed a remarkable hepatomegaly. Other positive findings were retarded bone age and elevated liver enzymes. He was referred to the pediatric gastroenterology section with a possible diagnosis of “GSD.” Where the parents refused to take a liver biopsy and the kid was discharged taking cornstarch four times a day, which was used for only 3 months. There were no further episodes of seizure or laboratory findings of hypoglycemia, metabolic acidosis, elevated blood lactate, uric acid, and hyperlipidemia in his outpatient follow-up visits.

The proband (IV-1), an 8-month old boy (Figure 2) was a result of a consanguineous marriage. There was no family history of inherited metabolic disease. At birth, he was 2.8 kg weight and 50 cm in length but suffered from the poor weight (from 6 kg to 3.7 kg) in 8 months. He was involved with neonatal hyperglycemia, which was improved spontaneously in a few days. Then during the first few months of life, he experienced failure to thrive, protruding abdomen, and polyuria, and clinical manifestations of rickets including widening of the wrist and bowing of the lower limbs. During infancy and early childhood, he was admitted to hospital several times due to severe growth retardation, episodes of lethargy, poor feeding, and diarrhea. Radiologic studies were in favor of rickets. Ultrasonography showed hepatosplenomegaly and enlargement of kidneys. Laboratory findings revealed normal anion gap, hyperchloremic metabolic acidosis, fasting hypoglycemia, and post-feeding hyperglycemia, hypokalemia, hypomagnesemia hypophosphatemia, elevated hepatic transaminases (ATS/ALT), proteinuria, glucosuria, hyperphosphaturia, and non-specific aminoaciduria. Analysis of liver biopsy samples revealed liver steatosis and glycogen accumulation in the hepatocytes.

The 11-year-old girl [proband (V-3), Figure 2] was the result of a kinship marriage with no family history of metabolic disorders. Her weight at birth was 3.2 kg. According to her past medical history, she was well in a few early months, but during the second half of the first year of life severe growth retardation, failure to thrive, hepatomegaly, and episodes of fever and lethargy gradually appeared. There was a history of the retarded cutting of the teeth, but no history of seizures, motor or cognitive decline, and the speech and social skills were within the normal ranges for age. She developed bone pain and walking difficulties following a gradual progressive deformity of the legs as the clinical manifestation of rickets and its complications since 10 months old. At the age of 2, she weighed only 2.8 kg and was involved with a severe failure to thrive and complicated rickets. Her main clinical problems in 11 years bold were dwarfism with skeletal deformities, protuberant abdomen due to hepatomegaly, moon-shaped face, fat deposition about the shoulders and abdomen, delayed puberty, osteoporosis with bone pain, and increased risk of pathologic fractures. Laboratory assessment showed hypoglycemia, ketonuria, glucosuria, proteinuria, increased transaminases (ATS/ALT), reduced serum phosphorus, and markedly elevated serum alkaline phosphatase levels. Normal serum calcium and decreased vitamin D levels were also recorded. Clinical and laboratory findings for the patients are reported in Table 1.

**TABLE 1:** Clinical and laboratory findings for the patients.

Patient no.	Patient 1 (Proband IV-2)	Patient 2 (Proband IV-1)	Patient 3 (Proband V-3)
GSD type	GSDVI	GSDXI	GSDXI
Mutation	PYGL	SLC2A2	SLC2A2
cDNA	c.298_307del	c.613-1G > C	c.1043dupT
Protein	p.M100Sfs*18		p.N349Kfs*44
Gender	M	M	F
Age(years)	5	9	11
Age at onset	3 years	4 months	5 months
Age at diagnosis	4 years	8 months	1 year
Initial presentations	Jaundice at birth, hepatomegaly, growth retardation, elevated liver enzymes	Lethargy, growth retardation, FTT, hepatomegaly, diarrhea, polyuria	Fever, lethargy, growth retardation, FTT, hepatomegaly
Liver biopsy	NA	Compatible aspect with GSD	NA
Hepatomegaly	+	+	+
Hypoglycemia (<60 mg/dl)	–	+	+
Increased AST/ALT (Up to 37 U/L)/(up to 41 U/L)	+/+	+/+	+/+
Hyperlipidemia (>160 mg/dl)	–	–	–
Hypercholesterolemia (>200 mg/dl)	–	–	–
Hyperlactatemia (>2.5 mg/dl)	–	–	–
Hyperuricemia (>5 mg/dl)	–	–	–
Proteinuria	–	+	+
Glucosuria	–	+	+
Ketonuria	–	–	+
Ammonemia	–	+	NA
Hypophosphatemia (< 4 mg/dl)	–	+	+
Increased alkaline phosphatase (>1200 U/L)	–	–	+
Serum calcium (8–13 mg/dl)	NA	Decreased	Normal
Vit D deficient (<20 ng/ml)	Normal	Normal	Decreased
Other features	–	-Rickets -Anemia	- Rickets

### Whole-Exome Sequencing Results

A summary of data analysis from the WES of the patients is shown in Table 2. WES on proband (IV-2) was generated 7.4 GB sequences. The average depth of target regions reached 124X before mapping to the human reference genome sequence (hg19) and after mapping, the average depth of target regions reached 83X. Coverage data showed that 98.6% of the target regions were sequenced at a more than depth of 10X reads. WES of the second patient (proband IV-1) was produced 5.6 GB sequences and over 55 million read pairs (101 × 2) were mapped. The mean depth of target regions reached 93X before mapping to the reference genome and after mapping, the mean depth of target regions reached 65X. Coverage data presented that 97.5% of the target regions were sequenced at a minimum depth of 10X reads. The quality control of 7.3 GB of the third patient (proand V-3) revealed 97.8% of the read bases had Q30. The throughput coverage of target regions extended 121X before mapping to the reference genome and after mapping, the mean depth of target regions was 77X. Furthermore, the analysis of sequencing data showed that 96.3% of the target regions were covered at a minimum depth of 10X reads.

**TABLE 2:** The summary report of data analysis from the WES.

Analytical characteristic	Patient 1 (Proband IV-2)	Patient 2 (Proband IV-1)	Patient 3 (Proband V-3)
Total number of reads	74,227,000	55,298,288	72,367,238
Average read length (bp)	101	101	101
Target Region (Mbp)	60	60	60
% Bases QV>30	98.26	98.59	98.15
% Initial Mappable Reads	99	99	99
% Minimum coverage of target regions (for depth 1X, 5X and 10X)	99.8, 99.4 and 98.6	99.7, 99.2 and 97.4	97.8, 97.3 and 96.3
% of duplicate reads (pre-alignment)	24	20.5	29.24
% of duplicate reads (post-alignment)	6	5	8.7
% On Target Reads (post-alignment)	72	70	77
% Coverage >25X	91.34	81.77	88.41
% Coverage >50X	62.97	45.94	60.94
# of SNV	126,424	117,459	117,960
# of Stop Gained	90	104	91
# of Indels	12,603	10,527	11,116

### Genetic Findings

The filtering steps of the variants were performed based on allele frequency < 0.05, mode of inheritance (keyword search: autosomal recessive variants in GSD-associated genes), pathogenic impacts of variants; such as CADD-PHRED score > 10, mutation taster (disease-causing), pathogenic scores in DANN Score (the value range is 0–1), and conservation prediction tools such as GERP-score (ranging from −12.3 to 6.17). Finally, our filtering strategy revealed three novel homozygous causative variants (two frameshift variants and one splice site variant) in three GSD suspected patients. The first frameshift variant, c.298_307delATGATCAACC (the 10 bp deletion), in exon 2 of PYGL gene was identified in patient 1 (proband IV-2). The second frameshift variant, c.1043dupT, in exon 7 of SLC2A2 gene was found in patient 3 (proband V-3). One novel splicing variant c.613-1G > C, at end of the fifth intron of SLC2A2 gene was identified in patient 2 (proband IV-1) as a homozygous variant. Multiple lines of *in silico* analysis were used to evaluate the pathogenicity of the variants which are shown in Table 3. Sanger sequencing was used to confirm the WES results. The identified causative variants in patients were homozygote state, and unaffected parents were found to be heterozygote state for the recognized variants (Figure 2). Other detected variants (including eight mutations) in three patients were secondary findings in Mendelian disease genes from the OMIM database (data are available upon request). No pathogenic/likely pathogenic variants have been found in the ACMG’s list of 59 actionable genes. The assessment of WES analysis revealed 10 pharmacogenetic variants that were at PharmGKB 1A/1B levels (Table 4).

**TABLE 3:** The variant characterization of identified variants such as computational analysis and variant frequency in population databases.

Patients	Patient 1 (proband IV-2)	Patient 2 (proband IV-1)	Patient 3 (proband V-3)
**Variant definition** -Gene name -Varian name -Protein change -Chromosome position (GRCh37) - Zygosity	PYGL (NM_002863) c.298_307del p. M100Sfs*18 Chr14:51404492-51404501 Homozygote	SLC2A2 (NM_000340.2) c.613-1G > C Chr3: 170723895 Homozygote	SLC2A2 (NM_000340.2) c.1043dupT p.N349Kfs*44 Chr3:170720390-170720391 Homozygote
***In silico* predictive tools** -CADD (Phred Score) -DANN -GERP -MutationTaster -HSF	33 (Deleterious) – 5.7 Disease-causing –	27.4 (Deleterious) 0.9933 (Deleterious) 5.51 Disease causing Propably affecting splicing	29 – 5.15 Disease-causing –
**Population Databases** -1000 GP -ExACb -GenomAD -ESP	– – – –	– – – –	– – – –
Related phenotypes (OMIM number)	HERS disease/GDSVI (OMIM: 232700)	Fanconi-Bickel syndrome/GSDXI (OMIM: 232700)	Fanconi-Bickel syndrome/GSDXI (OMIM: 232700)
Variant classification (based ACMG guideline)	Pathogenic (PVS1, PM2, PP3, PP4)	Pathogenic (PVS1, PM2, PP3, PP4)	Pathogenic (PVS1, PM2, PP3, PP4)

**TABLE 4:** The identified pharmacogenetic variants at PharmGKB 1A/1B levels.

Pharmacogenetic variants (rsID)	Gene	Chromosome position (GRCh37)	RefSeq	Variant name	Variant type	MAF	PharmGKB category	Drugs (drug responses)	Diseases/phenotypes	Patients (zygosity)
rs4149056	SLCO1B1	chr12:21331549	NM_006446.5	c.521T > C p.V174A	Missense	0.13	1A	Simvastatin	Muscular diseases, myopathy, central core	Patient 1 (het) Patient 3 (het)
rs9934438	VKORC1	chr16:31104878	NM_024006.6	c.174-136C > T	Intronic	0.32	1B	Warfarin	Arteriosclerosis, heart diseases, hemorrhage, myocardial infarction, peripheral vascular diseases, pulmonary embolism, stroke	Patient 1 (het) Patient 2 (het)
rs1135840	CYP2D6	chr22:42522613	NM_000106.6	c.1457C > G p.T486S	Missense	0.45	1A/1B	Paroxetine, nortriptyline, codeine, Doxepin, clomipramine, atomoxetine, amitriptyline, tramadol. Debrisoquine: (ultrarapid_ metabolism, clinvar drug-response)	Depressive disorder, major mental disorders obsessive-compulsive disorder, breast neoplasms, mood disorders, pain	Patient 1 (homo) Patient 2 (het) Patient 3 (homo)
rs35742686	CYP2D6	chr22:42524244	NM_000106.6	c.775delA p. R259Gfs*2	Frameshift	0.01	1A/1B	Paroxetine, codeine, doxepin, trimipramine, tamoxifen, nortriptyline, clomipramine, fluvoxamine, atomoxetine, amitriptyline, tramadol	Depressive disorder, major mental disorders obsessive-compulsive disorder, breast neoplasms, mood disorders, pain	Patient 1 (homo)
rs16947	CYP2D6	chr22:42523943	NM_000106.6	c.886T > C p. C296R	Missense	0.66	1A/1B	Paroxetine, tamoxifen, nortriptyline, fluvoxamine, codeine, doxepin, trimipramine, clomipramine, atomoxetine, amitriptyline, tramadol Debrisoquine-ultrarapid_metabolism (in clinvar drug-response)	Depressive disorder, major mental disorders obsessive-compulsive disorder, breast neoplasms, mood disorders, pain	Patient 1 (homo) Patient 2 (homo) Patient 3 (homo)
rs3745274	CYP2B6	chr19:41512841	NM_000767.5	c.516G > p.Q172H	Missense	0.272	1A	Efavirenz Methadone (in clinvar drug-response)	HIV infections	Patient 3 (het) Patient 2 (het)
rs7294	VKORC1	chr16:31102321	NM_000106.6	c.408C > G p.V136 =	Synonymous	0.395	1B	Warfarin Phenprocoumon, warfarin, acenocoumarol, Vitamin_K-Dependent_Clotting_Factors (in clinvar drug-response)	-	Patient 3 (homo)
rs2228001	XPC	chr3:14187449	NM_004628.5	c.2815C > A p.Q939K	Missense	0.633	1B	Cisplatin	Osteosarcoma, testicular neoplasms, urinary Bladder Neoplasms	Patient 2 (het)
rs2279343	CYP2B6	chr19:41515263	NM_000767.5	c.785A > G p.K262R	Missense	0.129	1A	Efavirenz	HIV infections	Patient 2 (het)
rs1065852	CYP2D6	chr22:42526694	NM_000106.6	c.100C > T p.P34S	Missense	0.209	1A/1B	Paroxetine, tamoxifen, nortriptyline, fluvoxamine, codeine, doxepin, trimipramine, clomipramine, atomoxetine, tramadol	Depressive disorder, major mental disorders obsessive-compulsive disorder, breast neoplasms, mood disorders, pain	Patient 2 (het)

## Discussion

Glycogen storage diseases are a group of rare inherited metabolic disorders that have clinical, locus, and allele heterogeneity. WES has been recognized to be an effective method to identify the genetic deficiencies in hereditary/mendelian disorders such as metabolic disorders (Rabbani et al., 2014; Shakiba and Keramatipour, 2018). In the present study, we conducted WES and tried to interpret the results of the variant analysis in three patients with liver GSD suspected diagnosis. Exome analysis detected three causative variants in GSD-associated genes including c.298_307delATGATCAACC (at PYGL gene; associated with GADVI), c.613-1G > C, and c.1043dupT (at SLC2A2 gene; associated with GSDXI). Computational analysis and variant frequency in population databases are shown in Table 3.

The detected variants in this study, frameshift, and splice site variants are null alleles (loss-of-function mutations) which lead to premature termination codons (PTCs). PTCs frequently trigger non-sense-mediated decay (NMD) pathway as one of the central mechanisms of RNA surveillance to degrade the mRNA, so that no protein is prepared or a truncated protein is produced (Kurosaki and Maquat, 2016).

Regarding the two frameshift variants, p. M100Sfs^∗^18 in the PYGL gene and p. N349Kfs^∗^44 in the SLC2A2 gene caused by deletions of 10 nucleotides c.298_307delATGATCAACC and insertion of one nucleotide c.1043dupT, respectively. When several deleted or inserted nucleotides of a coding sequence are not a multiple of three, a shift in the reading frame is produced and so the original amino acid sequence is lost (Guttmacher and Collins, 2002).

In the PYGL gene, 10 base pairs deletion impose a change of methionine to serine at the 100th codon and leads to a new reading frame that causes premature stop codon after 18 base pairs. This change may produce a truncated protein with 116 aa residue.

Also, in the SLC2A2 gene, the insertion of one base pair changes asparagine to lysine at codon 349 of exon 7. This substitution leads to an early premature stop codon after 44 base pairs which produce a truncated protein with 391aa residue shorter than the normal protein with 542 aa. *In silico* prediction analysis Varsome (Kopanos et al., 2019) along with the manual examination of the ACMG-AMP 2015 guidelines (Richards et al., 2015) predicted that both frameshift variants are pathogenic: (1) Null variant (frameshift) in a gene where LOF is a known mechanism of disease (PVS1). (2) The absence of detected variants in any of the population databases (PM2). (3) Multiple prediction tools support a damaging effect on the gene or the protein (PP3). (4) The patient’s phenotype is highly specific for GSD with a single genetic etiology (PP4). These two variants in the PYGL and SLC2A2 genes were validated by Sanger sequencing in patients (as a homozygous variant) and their parents (as a heterozygous variant).

**FIGURE 3:**
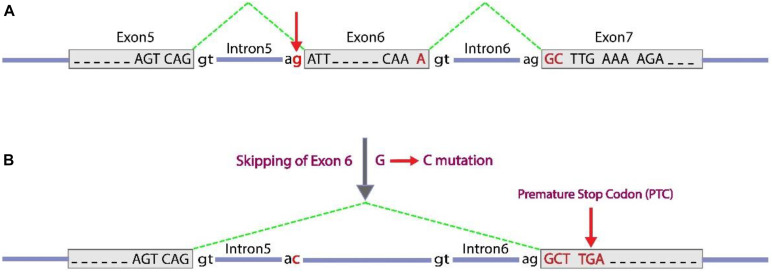
c.613-1G > C splice-site variant, can cause exon 6 skipping. Exon sequences are indicated with gray boxes and with capital letters to show nucleotides. Intron sequences are represented as blue horizontal lines with lower case letters to show key conserved nucleotides in splicing. The green dashed lines indicate the positions of splicing at the RNA level to bring together transcribed exon sequences. **(A)** A normal condition with three exons detached by introns with conserved terminal dinucleotides (5′ GT and 3′ AG, around an intron). **(B)** A substitution G to C occurred at the conserved 3′ terminal nucleotide of intron 5, which inactivate the splice acceptor site. As an alternative subsequent splice acceptor site of intron 6 could be used, which leads to skipping of exon 6 and following inducing a frameshift [lead to a premature stop codon (TGA or UGA at RNA level) as indicated].

Splice-site variants (canonical ± 1 or 2 splice sites) in major regulatory elements are located at or close to splice junctions often have significant effects on RNA splicing, especially if they alter highly conserved nucleotides, such as in GT or and AG dinucleotides at the 5′ and 3′ ends around any intron, respectively. These splice site variants cause loss of coding exon (by exon skipping or exon truncation) or a gain of coding exon sequences (by exon extension or intron retention) which may result in a translational frameshift at the RNA level. For the c.613-1G > C variant, possible consequences of a G to C substitution at the conserved 3′ splice-site consensus of intron 5 is to activate the splice acceptor site. The next splice acceptor site (at the 3′ end of intron 6) could be used instead, therefore, exon 6 will be skipped. As far as, the number of present nucleotides in exon 6 is not a multiple of three, so the change causes a frameshift in order of coding sequence. This frameshift induces PTC as illustrated in Figure 3. As mentioned in Section “Discussion,” *in silico* analyses of the effect of the c.613-1G > C variant on the splicing pattern and the strength (% values) of the acceptor splice site of exon 6 was predicted by the HSF program version 3^13^. Position weight (HSF) matrices predicted that the substitution of c.613-1G > C results in broken of wild type acceptor splice site with variation score (%) of wild type broken site −32 for HSF and −66 for MaxEnt (according to HSF and MaxEnt predictions, variation score under −10 and −30%, respectively, the mutation breaks the splice site and most probably affecting splicing; Desmet et al., 2009). Also, other prediction tools based on ACMG guidelines such as Varsome and Intervar tools indicated this variant is pathogenic based on this evidence: PVS1, PM2, and PP3.

In conclusion, our results indicate that the novel splice variant, c.613-1G > C, and the frameshift variant, p. N349Kfs^∗^44, in the *SLCA2* gene might be the genetic cause of FBS, and the other frameshift variant, p. M100Sfs^∗^18, in PYGL gene might be the genetic causes of GSD-VI. The recognized variants meet the criteria of interpreting the variants as being pathogenic, but we essentially advise applying functional analysis to study the distinctive pathological roles of the variants.

We also assessed patients’ data for incidental or secondary findings (in 59 genes listed in ACMG and OMIM genes) that are unrelated to the primary phenotype of each patient. Our findings and objectives can be categorized into two groups. First: The goal of reporting known pathogenic (KP) or expected pathogenic (EP) variants in the 59 gene list according to ACMG is to identify and determine the risks associated with highly penetrant genetic disorders over preventable or treatable interventions (Green et al., 2013; Kalia et al., 2017). Second: we also evaluate heterozygote pathogenic (P) and likely pathogenic (LP) variants in genes associated with mendelian and recessive disorders. This information on being heterozygote or carrier is important for genetic counseling and family follow-up genetic screening. In the present study, we did not identify any variant affecting the protein function of the list of 59 ACMG genes, but in our patients, we identified eight pathogenic/likely pathogenic heterozygous variants (data are available upon request). These variants in AR status may not be related to the patient’s phenotype, and so determining the possible carrier status of their parents for these variants is necessary before the next pregnancy. Therefore, appropriate genetic counseling and suitable schedules must be taken according to the genotype status of parents, for both their parents and even genetic screening of their relatives.

Analysis of pharmacogenetic variants unveils 10 variants reached a level 1A/1B classification in the PharmGKB database (extracted from pharmgkb.org) is shown in Table 4. To achieve more insight into the rare/common pharmacogenetic variations, we used accessible and easily available databases such as CPIC, PharmGKB, ClinVar, Pharmacogene Variation (PharmVar) Consortium. For example, the rs4149056 variant, with a MAF 0.13 in the GnomAD database, has a decreased function missense variant in the SLCO1B1 gene. This variant with heterozygous and homozygous states is accompanied by an intermediate and high myopathy risk, respectively, after getting simvastatin drug. If simvastatin is prescribed to a patient with intermediate SLCO1B1 function as a heterozygous variant, there is an increased risk for developing simvastatin-associated myopathy; such cases might require a lower starting dose of simvastatin or another statin agent (Ramsey et al., 2014). Two variants (rs9934438, rs7294) in VKORC1 gene have potential clinical utility. The genetic variances in VKORC1 gene are dependent on the difference in the final warfarin dose. Patients with the rs9934438 allele need a lower warfarin dose compared with the wild-type carriers (Wadelius et al., 2005). Patients with the rs7294 allele are prescribed a higher warfarin dose (Wadelius et al., 2005; Herman et al., 2006). Warfarin is a curative anticoagulant recommended for persons at the possibility of thrombosis and embolism (Owen et al., 2010).

However, in the local filtering of exome data, we detected both rare and common pharmacogenetic variants in a set of known genes in one research, our study has some limitations. We neglected some variants such as copy number variations (CNVs) and the majority of the intronic variants (as a result of lack of coverage for these regions in WES). Other technical issues may lead to undetected structural variants and the variants in highly homologous regions of the human genome (Hočevar et al., 2019). This may result in loosing some pharmacogenetic variants in cytochrome genes such as CYP2D6.

In summary, WES of the patients suspected to GSD diagnosis (with major symptoms such as hypoglycemia, hepatomegaly, and growth retardation) indicated three novel mutations associated with GSD-related genes. Two patients with early infantile age-onset had the causative variants in the SLC2A2 gene associated with GSD XI, while the patient with the early childhood age-onset had the pathogenic variant in the PYGL gene related to GSDVI. We hope that this identification may produce new insights into the mechanisms of pathogenesis of glycogen storage disorders. We also suggest doing functional analysis to evaluate the possible pathogenesis of the variants preceding to use in genetic counseling. Mutation detection in affected individuals helps to define the exact type of GSD and make the possibility of prenatal diagnosis (PND) and preimplantation genetic diagnosis (PGD) in families requiring this service. Here, we showed that WES technologies have the potentiality of identifying not only causative variants relevant to patients’ clinical manifestation but also revealed secondary/incidental findings in genes associated with AR disorders which have an impact on future generations and reproduction decisions. Moreover, secondary pharmacogenetics findings are important in personalized treatment or guiding therapy.

## Data Availability Statement

The raw data supporting the conclusions of this article will be made available by the authors, without undue reservation.

## Ethics Statement

The studies involving human participants were reviewed and approved by the Ethical Committee of the Tehran University of Medical Sciences. Written informed consent to participate in this study was provided by the participants’ legal guardian/next of kin. Written informed consent was obtained from the individual(s), and minor(s)’ legal guardian/next of kin, for the publication of any potentially identifiable images or data included in this article.

## Author Contributions

ME performed the experiments, and analysis and interpretation of the data, and drafting the manuscript. KF was involved in drafting the manuscript. MA was involved in drafting the manuscript and interpretation of the data. HS was involved in *in silico* analysis of datasets. ST, SS, and VY contributed to patients’ assessments and data collection. NO contributed to data collection from patients and molecular testing. MM designed and supervised the study and involved in drafting and finalizing the manuscript. All authors contributed to the article and approved the submitted version.

## Conflict of Interest

The authors declare that the research was conducted in the absence of any commercial or financial relationships that could be construed as a potential conflict of interest.

## Footnotes

1 http://www.hgmd.cf.ac.uk/ac/index.php

2 https://www.broadinstitute.org/gatk/

3 http://wannovar.wglab.org

4 http://www.ncbi.nlm.nih.gov/snp

5 http://browser.1000genomes.org

6 http://evs.gs.washington.Edu

7 http://exac.broadinstitute.org

8 https://gnomad.broadinstitute.org

9 http://www.ncbi.nlm.nih.gov/clinvar

10 http://www.hgmd.org

11 http://bioinfo.ut.ee/primer3–0.4.0/

12 https://www.ensembl.org

13 http://www.umd.be/HSF3/
